# Performance and Mechanism of As(III/V) Removal from Aqueous Solution by Fe_3_O_4_-Sunflower Straw Biochar

**DOI:** 10.3390/toxics10090534

**Published:** 2022-09-11

**Authors:** Yuling Zhao, Hao Shi, Xin Tang, Daihong Kuang, Jinlong Zhou, Fangyuan Yang

**Affiliations:** 1 College of Resources and Environment, Xinjiang Agricultural University, Urumqi 830052, China; 2 College of Mathematics and Physics, Xinjiang Agricultural University, Urumqi 830052, China; 3 College of Hydraulic and Civil Engineering, Xinjiang Agricultural University, Urumqi 830052, China

**Keywords:** As(III/V), sunflower straw biochar, Fe_3_O_4_, magnetic composite, adsorption

## Abstract

Humans and ecosystems are severely damaged by the existence of As(III/V) in the aquatic environment. Herein, an advanced Fe_3_O_4_@SFBC (Fe_3_O_4_-sunflower straw biochar) adsorbent was fabricated by co-precipitation method with sunflower straw biochar (SFBC) prepared at different calcination temperatures and different SFBC/Fe mass ratios as templates. The optimal pH for As(III/V) removal was investigated, and Fe_3_O_4_@SFBC shows removal efficiency of 86.43% and 95.94% for As(III) and As(V), respectively, at pH 6 and 4. The adsorption effect of calcining and casting the biochar-bound Fe_3_O_4_ obtained at different temperatures and different SFBC/Fe mass ratios were analyzed by batch experiments. The results show that when the SFBC biochar is calcined at 450 °C with an SFBC/Fe mass ratio of 1:5, the adsorption of As(III) and As(V) reaches the maximum, which are 121.347 and 188.753 mg/g, respectively. Fe_3_O_4_@SFBC morphology, structure, surface functional groups, magnetic moment, and internal morphology were observed by XRD, FTIR, SEM, TEM, and VSM under optimal working conditions. The material shows a small particle size in the range of 12–14 nm with better magnetic properties (54.52 emu/g), which is suitable for arsenic removal. The adsorption mechanism of As(III/V) by Fe_3_O_4_@SFBC indicates the presence of chemisorption, electrostatic, and complexation. Finally, the material was used for five consecutive cycles of adsorption–desorption experiments, and no significant decrease in removal efficiency was observed. Therefore, the new adsorbent Fe_3_O_4_@SFBC can be efficiently used for arsenic removal in the aqueous system.

## 1. Introduction

Arsenic is commonly present in the aquatic environment. Arsenic-contaminated water bodies affect populations in most parts of the world, including Mexico, Argentina, China, India, and Bangladesh [1,2]. A total of 2.5 million people living in specific areas of China have greater than 50 μg/L arsenic in drinking water [3]. Prolonged exposure to arsenic in water may cause an increased risk of skin cancer, lung cancer, and esophageal cancer in humans [4]. Hence, the World Health Organization established a 10 μg/L upper limit in drinking water for total arsenic [5]. Therefore, it is important to prepare effective wastewater treatment methods to reduce the health risks associated with arsenic water pollution.

Among these methods, membrane separation [6], ion exchange [7], and bioremediation [8], adsorption are potentially effective for arsenic removal due to their lower cost, highly efficient, convenient, and eco-friendly processes [9]. Until now, multiple adsorbents, including activated carbon [10], clay mineral materials [11], metal oxides/hydroxides [12], natural organic compounds [13], and nanocomposites [14], have been investigated as media for arsenic removal.

In recent years, nanocomposite adsorbents have received increasing attention due to their specific high surface area and excellent reactivity via abundant adsorption sites [15]. There is no doubt that high adsorption and selectivity are key points for materials with adsorption potential. According to Awual et al. [16,17], composite materials are of increasing interest for arsenic removal based on their specific functionality and surface area. Among them, Fe_3_O_4_ nanomaterials with superparamagnetic characteristics make the separation of adsorbents easy from the aqueous phase under the influence of an applied magnetic field [18]. In addition, these materials exhibit good heavy metal adsorption properties [19]. However, nanoscale Fe_3_O_4_ materials are highly susceptible to oxidation to non-magnetic materials and loss of dispersibility due to their high chemical activity [20,21]. Ways to overcome these defects and structural modifications are needed to prevent aggregation.

Biochar is a porous, carbonaceous material obtained by the pyrolysis of biomass waste under anaerobic/limited oxygen conditions [22]. New biochar-based adsorbents can be used as environmentally friendly carriers for metal oxide dispersion. Thus far, hydroxides or oxides of metals such as Pb, Cu, Cd, Zn, Fe, Bi Al, Mn, Ce, La, etc., have been fabricated as As(V) adsorbents [23,24]. An amount of 90 mg/g was the highest As(V) adsorption capacity determined by Wen et al. [25], using a nano-biochar composite of magnetically active tea waste. Iron oxide nanoneedles were deposited on a biochar surface made of cotton fiber which increased the adsorption capacities of As(V) and As(III) to 93.94 and 70.22 mg/g, respectively. Furthermore, Wang et al. [26] used pine biochar as zero-valent iron-loaded particles with 124.5 mg/g adsorption capacity for As(V).

Sunflower straw is often used for preparing livestock feed and composting fertilizers; as an agricultural by-product, its yield is high, and its cost is low. Additionally, the adsorption capacity of using it in combination with Fe_3_O_4_ as a nano sorbent is yet to be investigated, and its possible removal mechanism is not clear. Therefore, the aim of this work was mainly to prepare a cleaner and recyclable Fe_3_O_4_-biochar composite (Fe_3_O_4_@SFBC)using sunflower straw (SFBC) for efficient As(III/V) removal from water. The effects of different calcination temperatures (350, 450, 550, and 650 °C) and Fe_3_O_4_ materials prepared with different SFBC/Fe ratios (1:2, 1:3, 1:4, 1:5, and 1:6) on the As(III/V) adsorption, respectively, were studied. Adsorption studies with varying pH, initial concentration, and reaction time were also conducted to obtain the optimal adsorption conditions, while the adsorption isotherms and kinetics were studied to explore the adsorption process. In addition, adsorption–desorption experiments were conducted to measure the performance of the material for As(III/V) adsorption in terms of low cost and ease of application.

## 2. Materials and Methods

### 2.1. Materials

Sunflower stalks were harvested through local fields in Bayannur, in Inner Mongolia, China. Before use, stalks were rinsed twice with tap water, washed three times using deionized water to eliminate impurities, dried in air for 24 h, and then dried thoroughly in an oven at 80 °C. The dried sunflower stalks were crushed, sieved, and sealed for later use. Two iron salts (FeCl_3_·6H_2_O and FeSO_4_·7H_2_O) and ammonia water (NH_3_·H_2_O) were purchased from Tianjin Zhiyuan Chemicals. Deionized water was used for solution preparation. Analytical grade reagents were used without any further purification.

### 2.2. Sunflower Straw Pretreatment

The crushed sunflower straws were positioned in a 30 mL covered porcelain crucible and heated at 350, 450, 550, and 650 °C under anaerobic conditions. The heating rate was 5°/min for 2 h. After calcination, the materials were washed twice with deionized water–anhydrous ethanol by centrifugation to remove fine particulate matter and water-soluble organic residues. Finally, the materials were oven dried at 80 °C. Synthesized materials were labeled as SFBC 350, SFBC 450, SFBC 550, and SFBC 650.

### 2.3. Synthesis of Fe_3_O_4_@SFBC Magnetic Nanoparticles

Different Fe_3_O_4_@SFBC nano absorbents material were prepared by co-precipitation method using SFBC 450 with an SFBC/Fe ratio of 1:5. First, 5.40 g and 2.78 g of FeCl_3_·6H_2_O and FeSO_4_·7H_2_O were taken and 100 mL deionized water added to dissolve these salts by ultrasound. Then, 1.62 g of SFBC 450 was poured into a beaker containing 10 mL of deionized water, and both liquids were mixed in a double-necked flask and de-oxygenated with a vacuum pump. The water bath was then heated to 90 °C, and 10 mL of NH_3_·H_2_O was injected and stirred for 2 h, allowed to precipitate, followed by cooling at room temperature. Centrifugation was performed for 5 min at 9000 rpm, and the precipitate was collected and washed twice using an alternating step of deionized water–anhydrous ethanol. The final product of Fe_3_O_4_@SFBC was obtained after drying under a vacuum for 12 h at 60 °C.

In addition, the material solutions prepared by different SFBC/Fe mass ratios (1:2, 1:3, 1:4, and 1:6) and biomass char at different calcination temperatures (350, 550, and 650 °C) were used to prepare different composites.

### 2.4. Characterization

The mineralogy of Fe_3_O_4_@SFBC was characterized using XRD (Bruker D2, Karlsruhe, Germany) with Cu Kα radiation in the range of 2θ = 5~80°. Morphology of biochar and nanomaterials was observed by using scanning electron microscopy (ZEISS GeminiSEM 300, Oberkochen, Germany) and transmission electron microscopy (JEM2100F, Akishima-shi, Japan). FTIR spectra of nanoparticles were measured using the KBr compression method from 400 to 4000 cm^−1^ with an infrared photo spectrometer (Scientific Nicolet iS5, Waltham, MA, USA). The magnetic behavior of the particles was studied using a vibrating sample magnetometer (MPMS-XL-7, San Diego, CA, USA) in the varying magnetic field strengths of ±2000 magnetic moments. The type and valence of the sample elements were determined with an X-ray electron spectrometer (ESCALAB 250XI, Waltham, MA, USA).

### 2.5. Adsorption Experiments

As(III/V) removal by Fe_3_O_4_@SFBC nanocomposites were investigated and optimized. Batch adsorption tests were conducted in the laboratory to optimize parameters for arsenic adsorption, such as pH, adsorption isotherm, and adsorption kinetics. An amount of 0.1 g adsorbent Fe_3_O_4_@SFBC was added to beakers containing 100 mL solution of As(III) and As(V) of 5 mg/L concentration, respectively. The solution was shaken for 1 h at room temperature under continuous stirring. The adsorbent was removed from the solution using an external magnet.

The parameters studied include solution pH, adsorption time, and initial As(III/V) concentration. In pH experiments, 0.1 mol/L of NaOH or HCl was used to adjust the pH. Generally, adsorption was performed in the pH range of 3–11. A total of 0.1 g of Fe_3_O_4_@SFBC nanoparticles were added to 100 mL of 5 mg/L As(III/V) solution, mixed, and stirred for some time. The samples were filtered by a 0.45 um membrane filter to collect filtrate and analyzed by an atomic fluorescence spectrophotometer (PF6-3, Beijing Purkinje General Instrument Co., Ltd., Beijing, China).

Before testing the samples using the atomic fluorescence spectrophotometer, the arsenic standard stock solution was used to configure the arsenic standard solution and draw the standard curve. After pretreatment, the sample enters the atomic fluorescence instrument through the sensor and generates arsine gas under the reducing acidic potassium borohydride conditions. The hydride forms ground state atoms in the argon–hydrogen flame, and its ground state atoms are excited by the light emitted from the arsenic lamp to produce atomic fluorescence, and the intensity of atomic fluorescence is proportional to the element to be measured in the test solution under the standard curve.

The adsorption amount (*q_t_*, mg/g) of arsenic at the sampling time (min) and the adsorbent removal efficiency (*R*, %) were calculated by Equations (1) and (2), respectively.

(1) $$q_{t}=\frac{\left(C_{0}-C_{t}\right)}{m}V$$

(2) $$R(\%)=\frac{\left(C_{0}-C_{t}\right)}{C_{0}}\times 100\%$$

where *V* is the volume of arsenic solution (L), *C*_0_ is an arsenic concentration at an initial time and *C_t_* at time t (mg/L), and magnetic adsorbent mass (g) is denoted by *m*.

### 2.6. Desorption and Regeneration Texts

The adsorption–desorption cycle for each adsorbent was studied for five cycles. In each cycle, 5 mg/L of As(III) or As(V) solution was mixed with 1 g/L of adsorbents for 1 h on a magnetic stirrer, and desorption of As was carried out by mixing with 200 mL NaOH [27] with a concentration 1 mol/L for 30 min. Washing of the adsorbent was carried out by 0.01 mol/L HCl solution until the solution pH turned neutral. Finally, the Fe_3_O_4_@SFBC nanoparticles were dried in an oven for 2 h at 60 °C under a vacuum. Nanoparticles were collected with an external magnet for the next cycling experiments.

## 3. Results

### 3.1. XRD and FTIR Spectroscopy Analysis

**Figure 1:**
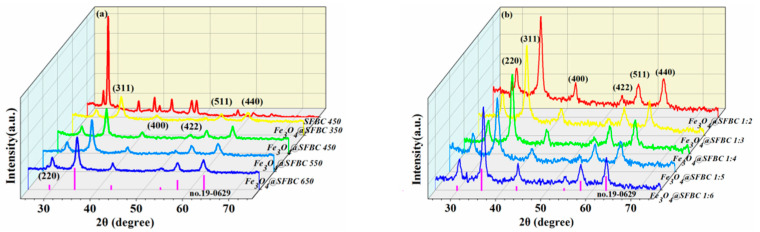
XRD spectra of Fe_3_O_4_ nanomaterials prepared by biochar at different calcination temperatures (**a**) and XRD spectra of Fe_3_O_4_ nanomaterials prepared by different SFBC/Fe mass ratios (**b**).

In order to evaluate the crystalline structure, an X-ray diffraction analysis of the synthesized Fe_3_O_4_@SFBC was carried out. Figure 1a shows the XRD patterns of Fe_3_O_4_@SFBC nanoparticles synthesized at different pyrolysis temperatures (SFBC/Fe mass ratio of 1:5), and Figure 1b reveals the XRD patterns of Fe_3_O_4_@SFBC nanoparticles produced at different SFBC/Fe mass ratios (biochar calcination temperature of 450 °C). The distinctive peaks of all samples were concentrated in the 2θ range of 30.4°, 35.6°, 43.3°, 53.7°, 57.4°, and 62.9°; agreeing with (400), (422), (220), (311), (511), and (440) crystal planes; and matching the Fe_3_O_4_ standard card (JCPDS card no. 19-0629). These patterns indicate that the samples are all Fd-3m counter spin Fe_3_O_4_ single crystals [28]. The SFBC in the Fe_3_O_4_@SFBC magnetic composite structure cannot alter the structure of Fe_3_O_4_. The main peak of SFBC 450 is located at 29.57°main peak (Figure 1a) matches the amorphous structure diffraction peak, linked to the crystalline structure of cellulose in the biomass structure. Additionally, metal salts affect biomass char yield. Additionally, the main peak, K, Ca, and Si diffraction peaks, are also present in SFBC 450 [29]. However, this sharp peak is found only in the nanoparticles of Fe_3_O_4_@SFBC 1:2 with the highest SFBC content, and the peak at 29.57° is not noted for the other samples, attributed to the high intensity of Fe_3_O_4_ crystals in other samples with a high strong crystallinity that masks the presence of amorphous structures [30].

**Figure 2:**
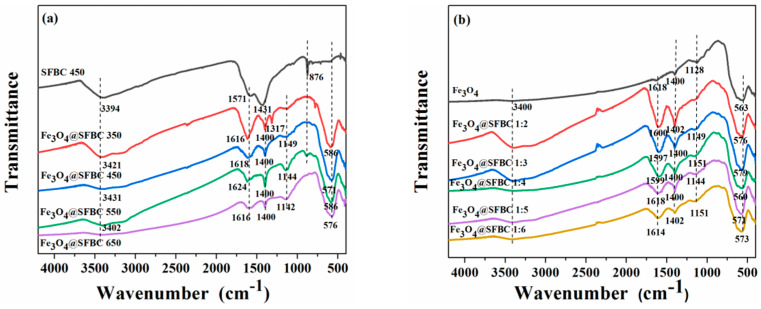
FTIR spectra of Fe_3_O_4_ nanomaterials prepared using biochar at different calcination temperatures (**a**) and FTIR spectra of Fe_3_O_4_ nanomaterials prepared by different SFBC/Fe mass ratios (**b**).

Figure 2a shows the FTIR patterns of Fe_3_O_4_@SFBC nanoparticles prepared by biochar synthesized at different pyrolysis temperatures (SFBC/Fe mass ratio of 1:5), and Figure 2b indicates the FTIR patterns of Fe_3_O_4_@SFBC nanoparticles manufactured according to diverse SFBC/Fe mass ratios (biochar calcination temperature of 450 °C). In both graphs, the broad peak observed around 3400 cm^−1^ denotes the hydroxyl group present in the sample. The FTIR spectra of Fe_3_O_4_@SFBC exhibit two peaks at 1634 cm^−1^ and 1200 cm^−1^ for bending of water and C-O stretching, respectively [31]. The peak at around 1400 cm^−1^ is associated with C-C and -COOH bond vibrations [32], and that bond vibration peak approves the formation of the carbonaceous structure of the composite. The peaks at about 583 cm^−1^ and 1140 cm^−1^ in the Fe_3_O_4_@SFBC spectra are the Fe-O stretching vibration peak and Fe-OH absorption peak [33]. The relative intensities of C-C, C-O, and Fe-O groups are, to some extent, altered as compared with the original SFBC and Fe_3_O_4_, demonstrating the successful synthesis of Fe_3_O_4_@SFBC nanoparticles. The peak intensities of the eight materials in both graphs are attributed to the different SFBC calcination temperatures and the mass ratios.

### 3.2. Arsenic Removal Experiments

#### 3.2.1. Effect of pH of the Solution

According to previous studies, pH can affect the adsorption capacity and efficiency by influencing surface loading and ionization extent of different functional groups [34]. Hence, the adsorption efficiency of As(III/V) was determined by Fe_3_O_4_@SFBC (when the SFBC biochar is calcined at 450 °C with an SFBC/Fe mass ratio of 1:5) from the pH range of 3 to 11.

**Figure 3:**
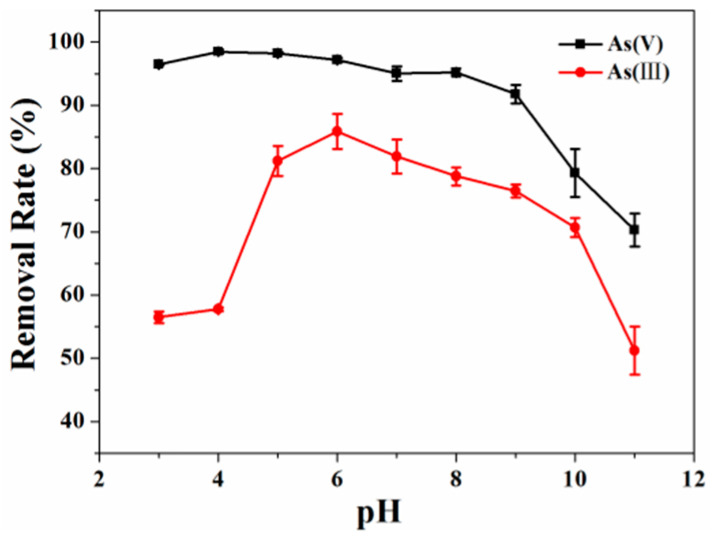
Effect of pH on the removal of As(III/V) (experimental conditions: pH = 3–11, dose = 1 g/L, initial concentration = 5 mg/L, T = 25 °C), *Y* error bars indicate the standard deviation of each data point (*n* = 3).

The initial pH effect on As(III/V) adsorption by Fe_3_O_4_@SFBC is shown in Figure 3. In brief, a change in the optimal pH of As(III/V) by Fe_3_O_4_@SFBC was observed, which is 6 and 4, respectively. The pKa1, pKa2, and pKa3 of H_3_AsO_3_ are 9.1, 12.1, and 13.4, respectively. The pKa1, pKa2, and pKa3 of H_3_AsO_4_ are 2.1, 6.7, and 11.2. Under most pH conditions, As(V) is present in a negative ionic form (H_2_AsO_4_^-^ and HAsO_4_^2-^), whereas As(III) is in a nonionic form (H_3_AsO_3_).

The rate of removal of As(III) increased directly with pH until pH 6 and decreased inversely with pH at greater than pH 6. This is attributed to the availability of As(III) in neutral form (H_3_AsO_3_) from pH 3 to 9. For As(III), the number of negatively charged arsenic species increases with increasing pH. However, the adsorption of As(III) on Fe_3_O_4_@SFBC composites did not decrease unidirectionally. The increase in the adsorption of As(III) in neutral solutions suggests that its adsorption process on Fe_3_O_4_@SFBC is followed by surface complexation rather than electrostatic interactions [35].

Moreover, greater As(V) removal efficiency is observed at low pH, while it decreases significantly at higher pH values. At pH 4, the maximum removal rate is 95.94%; at pH 11, it changes sharply to 67.67%. In general, the adsorption of As(V) on Fe_3_O_4_@SFBC composites is mainly governed by electrostatic attraction [36]. At smaller pH values, positively charged H^+^ in the water leads to the strong electrostatic attraction between arsenate anions (HAsO_4_^2−^ and AsO_4_^3−^) and the positively charged adsorbent surface. However, at greater pH values, maximum -OH concentration makes the adsorbent negative charge, generating machine repulsion [37].

#### 3.2.2. Adsorption Kinetics

The influence of contact time on As(III/V) adsorption was evaluated by atomic fluorescence spectrometry. A total of 0.5 g Fe_3_O_4_@SFBC was added to a 500 mL solution of 5 mg/L As(III) or As(V) solution, and pH 6 or 4 were maintained for As(III) or As(V) with contact time from 0 to 150 min. Samples were collected after adsorption, followed by filtration through a 0.45 μm membrane. The solids were dried for subsequent characterization. The adsorption process of Fe_3_O_4_@SFBC on arsenic were revealed by kinetic experiments. Models of pseudo-first-order (Equation (3)) and pseudo-second-order (Equation (4)) used are given below:

(3) $$\ln\left(q_{e}-q_{t}\right)=\ln q_{e}-k_{1}t$$

(4) $$\frac{t}{q_{t}}=\frac{1}{k_{2}{q_{e}}^{2}}+\frac{t}{q_{e}}$$

where *t* is the equilibration period (min), *q_t_* and *q_e_* are the adsorption amounts at time t and equilibrium (mg/g), respectively, *k*_1_ (min^−1^) and *k*_2_ (g/(mg·min)) are the rate constants for pseudo-first-order and pseudo-second-order kinetics, respectively.

**Figure 4:**
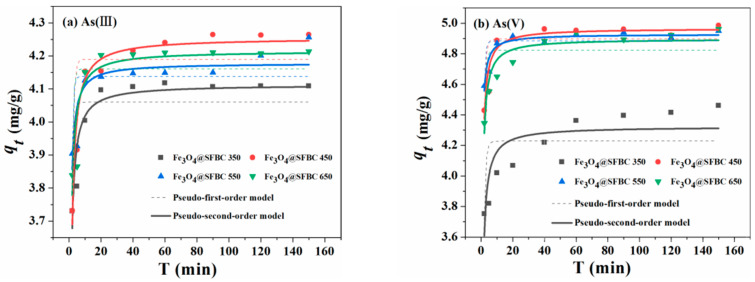
Adsorption kinetics of As(III) (**a**) and As (V) (**b**) on Fe_3_O_4_@SFBC (prepared from biochar at different calcination temperatures) (experimental conditions: pH = 6 and 4, dose = 1 g/L, initial concentration = 5 mg/L, T = 25 °C).

**Figure 5:**
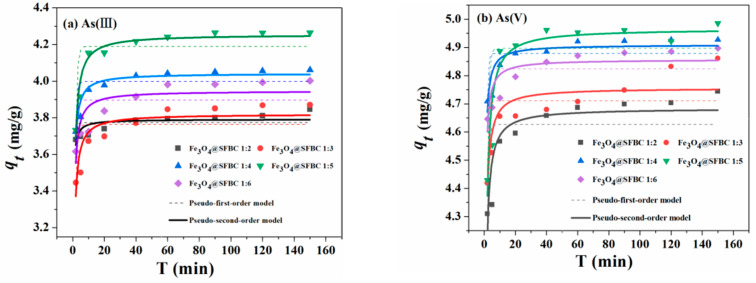
Adsorption kinetics of As(III) (**a**) and As(V) (**b**) on Fe_3_O_4_@SFBC (different SFBC/Fe mass ratios) (experimental conditions: pH = 6 and 4, dosage = 1 g/L, initial concentration = 5 mg/L, T = 25 °C).

Figure 4 and Figure 5 show the effect of contact time on As(III/V) adsorption of Fe_3_O_4_@SFBC prepared from biochar with different calcination temperatures and SFBC/Fe with different mass ratios. Regarding the adsorption of As by the above materials, compared to the pseudo-first-order, the experimental findings agree well with the pseudo-second-order. Obtained kinetics calculations are presented in Table 1. The higher value *R*^2^ of the pseudo-second-order kinetics for the Fe_3_O_4_@SFBC nanomaterials indicates the experimental data are better and more accurate [38]. This also suggests that the process of adsorption follows chemisorption [39]. Furthermore, Figure 4 and Figure 5 show that the adsorption of arsenic by Fe_3_O_4_@SFBC nanomaterials is a time-dependent process.

#### 3.2.3. Adsorption Isotherm

Maximum adsorption of As(III/V) by Fe_3_O_4_@SFBC is evaluated by an adsorption isotherm. Initially, As(V) or As(III) solution concentrations are maintained at 0.1, 0.5, 1, 3, 5, 7, and 10 mg/L with same pH value as in kinetic experiment. The 0.1 g of Fe_3_O_4_@SFBC was mixed with 100 mL arsenic solution followed by 1 h shaking at constant temperature (25 °C). It was then placed in an external magnet for the separation of magnetic nanoparticles. A membrane filter was used for supernatant filtration and analyzed by atomic fluorescence spectrophotometer to assess the residual arsenic concentration. In order to study the adsorption behavior of As(III/V) after Fe_3_O_4_@SFBC, the Langmuir model (Equation (5)) and Freundlich model (Equation (6)) were used for fitting the experimental data.

(5) $$q_{e}=\frac{q_{m}K_{L}C_{e}}{1+K_{L}C_{e}}$$

(6) $$q_{e}=K_{f}C_{e}^{1/n}$$

where *C_e_* indicates As concentration (mg/L) at equilibrium, *q_e_* is the adsorption capacity (mg/g) at adsorption equilibrium, and *q_m_* represents the maximum adsorption capacity at single-molecule layer adsorption (mg/g). *K_L_* is the Langmuir constant linked with the thermodynamics of the adsorption process, and *n* and *K_f_* are the Freundlich constants.

**Figure 6:**
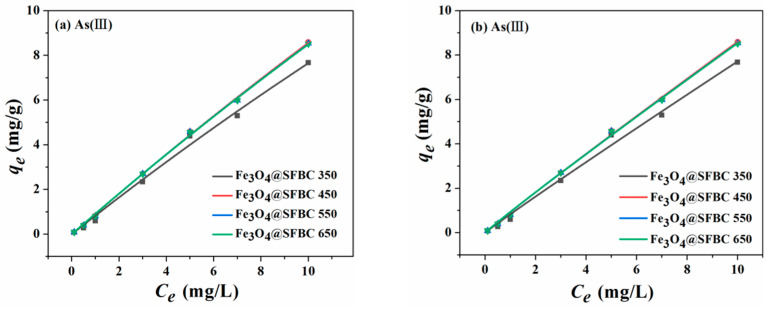
Adsorption isotherms of Langmuir (**a**) and Freundlich (**b**) on As(III) by Fe_3_O_4_@SFBC (prepared from biochar at different calcination temperatures) (experimental conditions: pH = 6, dose = 1 g/L, initial concentration = 0.1–10 mg/L, T = 25 °C).

**Figure 7:**
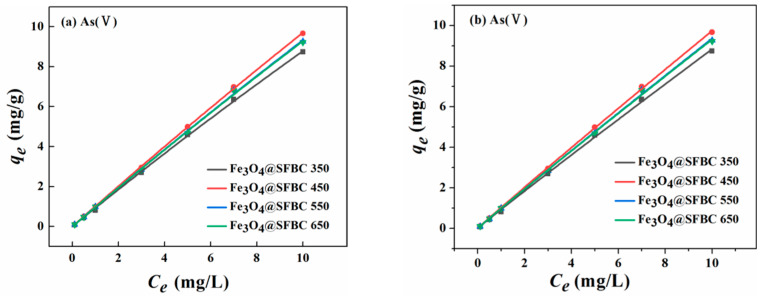
Adsorption isotherms of Langmuir (**a**) and Freundlich (**b**) on As(V) by Fe_3_O_4_@SFBC (prepared from biochar at different calcination temperatures) (experimental conditions: pH = 4, dose = 1 g/L, initial concentration = 0.1–10 mg/L, T = 25 °C).

**Figure 8:**
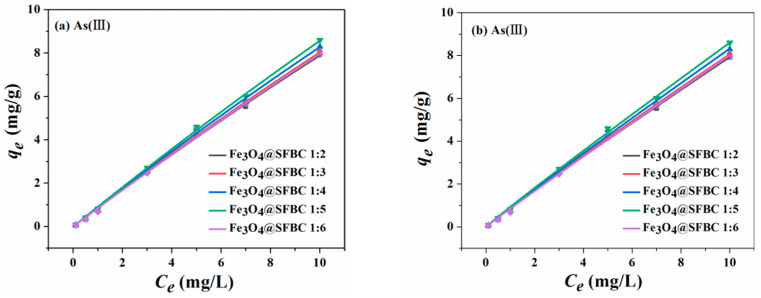
Adsorption isotherms of Langmuir (**a**) and Freundlich (**b**) on As(III) by Fe_3_O_4_@SFBC (different SFBC/Fe mass ratios) (experimental conditions: pH = 6, dose = 1 g/L, initial concentration = 0.1–10 mg/L, T = 25 °C).

**Figure 9:**
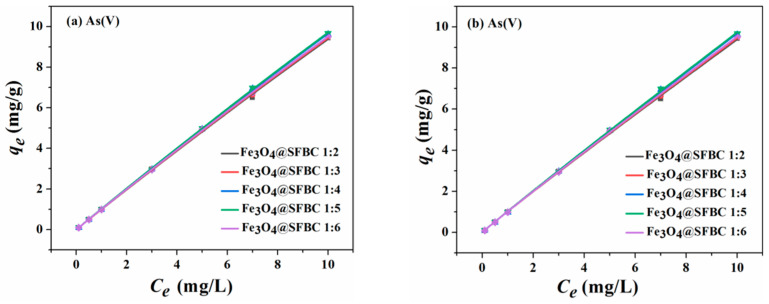
Adsorption isotherms of Langmuir (**a**) and Freundlich (**b**) on As(V) by Fe_3_O_4_@SFBC (different SFBC/Fe mass ratios) (experimental conditions: pH = 4, dose = 1 g/L, initial concentration = 0.1–10 mg/L, T = 25 °C).

The fitted curves of Langmuir and Freundlich are given in Figure 6, Figure 7, Figure 8 and Figure 9. The isotherm parameters are revealed in Table 2. For As(III/V), the Langmuir isotherm and Freundlich isotherm fit the experimental data well, and the coefficient of determination *R*^2^ does not differ significantly for both As(III/V).

Consequently, the removal of As(III/V) by Fe_3_O_4_@SFBC comprises both monolayer adsorption on a uniform surface and complex multilayer adsorption [40]. The optimal adsorption follows the Langmuir isotherm model based on the SFBC-modified Fe_3_O_4_@SFBC (SFBC/Fe of 1:5). The best adsorption system of Fe_3_O_4_@SFBC material prepared from SFBC material calcined at 450 °C according to the SFBC/Fe mass ratio of 1:5 was studied, and the absorption capacity of As(III) and As(V) is around 121.374 and 188.753 mg/g, respectively. Moreover, the adsorption of As(III/V) by Fe_3_O_4_@SFBC nanomaterials is greater than that of other adsorbents in previous articles (Table 3).

Biochar mostly contains elements such as C, H, O, and N, and its surface contains carboxyl, phenolic hydroxyl, carbonyl, anhydride, and other groups [41]. The physicochemical characteristics of biochar are closely linked to the pyrolysis temperature, and the pyrolysis process can be roughly divided into three temperature stages, 220–310 °C for hemicellulose pyrolysis and 315–400 °C for cellulose pyrolysis, and when the temperature exceeds 500 °C, the structure of biochar usually contains only an aromatic ring structure [42,43]. Moreover, the rise in the specific surface area of biochar with the increasing pyrolysis temperature and adsorption sites are useful for the adsorption of heavy metals; therefore, the temperature conditions should be controlled in the practical application to achieve the best results. These results suggest that the material prepared using biochar fired at 450 °C exhibits the best adsorption performance for the studied metal ions since it possesses more C=C and C-H functional groups at this temperature [44,45].

**Table 3:** Various adsorbent materials’ Langmuir adsorption capacities.

Fe-Biochar-Based Sorbents	Object	Maximum Adsorption Capacity (mg/g)	Reference
Fe_3_O_4_@SFBC	As(III)	121.374	This study
	As(V)	188.753	
Fe_3_O_4_@CSAC	As(III)	80.99	[46]
	As(V)	107.96	
Iron-modified activated carbon	As(V)	43.6	[47]
Iron(III) loaded orange waste	As(V)	68.6	[48]
Bioinspired 2D-carbon flakes-Fe_3_O_4_	As(III)	57.47	[49]
Fe-Zr-BC	As(III)	107.57	[50]
	As(V)	40.79	
magneticgelatin-modified biochar	As(V)	42.7	[51]

The use of optimal doses of biochar is essential to maximize arsenic removal from water. Biochar adsorption efficiency decreases with a high concentration above the optimum limit [22]. Therefore, changing the ratio of SFBC to iron ions in Fe_3_O_4_@SFBC material can improve optimal adsorption. This can be explained by the functional site saturation on the surface of biochar and partially due to available binding sites activation or pores on the biochar surface by the limited amount of Fe ions, which saturates the surface functional sites of the Fe_3_O_4_@SFBC material during the adsorption process.

### 3.3. Discussion of Morphology and Adsorption Mechanism

Fe_3_O_4_@SFBC (SFBC calcination temperature is 450 °C, SFBC/Fe is 1:5), which has the largest adsorption capacity, was taken as an example.

#### 3.3.1. Morphology

**Figure 10:**
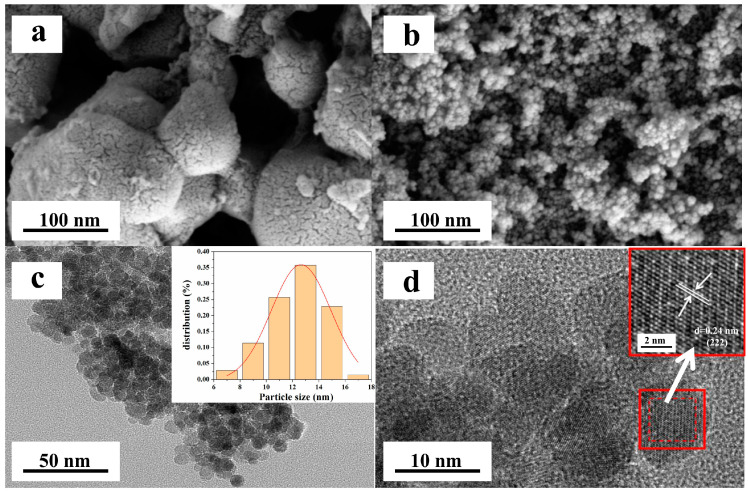
SEM images of SFBC (**a**) and Fe_3_O_4_@SFBC (**b**). TEM particle size distribution (**c**) and TEM lattice stripe (**d**) patterns of Fe_3_O_4_@SFBC.

The morphology of Fe_3_O_4_@SFBC was further examined by SEM and TEM. The surface of SFBC is fragmented, with uneven particle size and irregular surface shape, filled with porous structures and unevenness, as demonstrated in Figure 10a. SEM and TEM images of Fe_3_O_4_@SFBC show that Fe_3_O_4_ combines with SFBC biomass charcoal to form spherical nanoparticles of uniform size (Figure 10b,c). The histogram of the particle size achieved from the corresponding transmission electron microscopy image (upper right corner of Figure 10c) displays that in Fe_3_O_4_@SFBC, particle size is primarily homogeneously distributed in the range of 12–14 nm. The lattice stripe spacing diagram of Fe_3_O_4_@SFBC was obtained from Figure 10d with 0.24 nm a lattice gap, corresponding to the (222) crystal lattice plane in XRD, again confirming that the composition of the prepared sample is mainly Fe_3_O_4_ single crystal.

#### 3.3.2. Magnetic Properties

**Figure 11:**
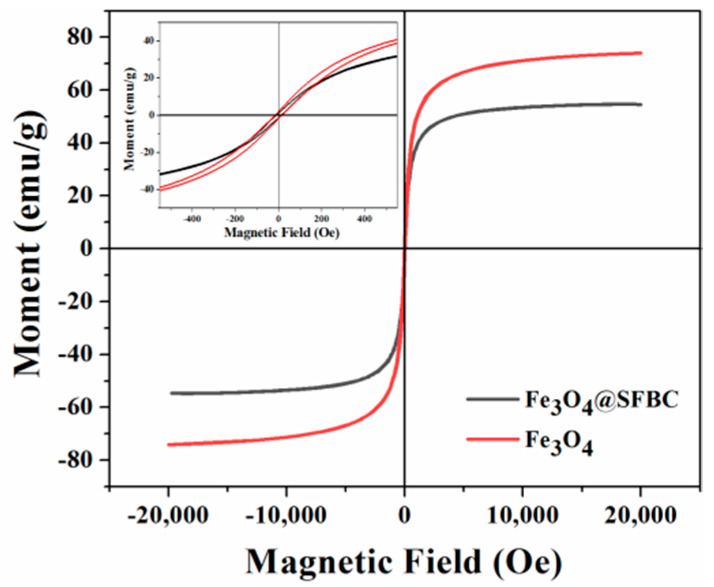
Magnetic hysteresis loop of Fe_3_O_4_ and Fe_3_O_4_@SFBC nanoparticles.

Magnetic strength is a key characteristic of magnetic adsorbents as it helps in the separation of the adsorbent from the aqueous solution and decreases the method cost. Fe_3_O_4_ nanoparticles and Fe_3_O_4_@SFBC magnetic nanocomposites were analyzed in the range of −2000 Oe to 2000 Oe, as shown in Figure 11. The saturation magnetizations (Ms) and magnetic remanence (Mr) values of Fe_3_O_4_ and Fe_3_O_4_@SFBC are 74.02 and 1.85, 54.52 and 1.22 emu/g, respectively, while the corresponding coercivity (Hc) values are 15.01 and 11.30 Oe. A non-magnetic matrix (SFBC) may be responsible for the reduction in magnetic saturation in the prepared magnetic nanocomposites.

#### 3.3.3. Adsorption Mechanism

The adsorption mechanism of As(III/V) using Fe_3_O_4_@SFBC was further evaluated by XPS. Changes in the chemical state of O, As, Fe, and C in Fe_3_O_4_@SFBC were determined before and after adsorption.

**Figure 12:**
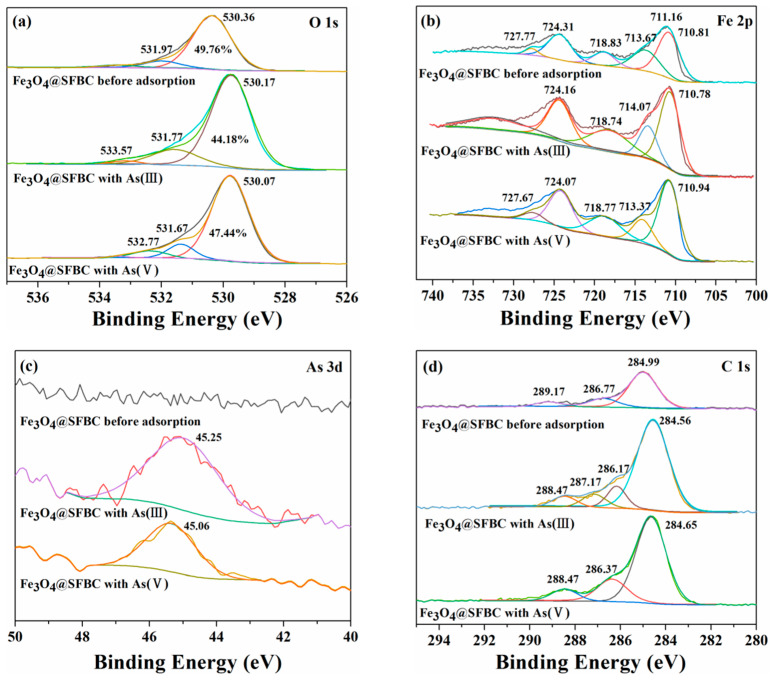
XPS spectra of Fe_3_O_4_@SFBC before and after arsenic adsorption: O 1s (**a**), Fe 2p (**b**), As 3d (**c**), C 1s (**d**).

Figure 12a shows three peaks appearing in the O 1s spectra at binding energies of 530, 531, and 532–533 eV, corresponding to lattice oxygen (O_2_), hydroxyl group, and adsorbed water in metal oxides, and the peaks at binding energies of 530.4 and 531.4 eV were assigned to Fe-O-H and Fe-O-C, respectively. These peaks are associated with the formation of the matching iron oxides during the adsorption of iron and oxygen. After adsorption of As(III/V), the main peak height of O 1s increased, attributed to the adsorption of arsenic by Fe_3_O_4_@SFBC (Figure 12a) [52]. The obtained findings show that the surface complexation between functional groups and heavy metals is complemented by the electrostatic attraction between O atoms and heavy metal ions.

Figure 12b depicts the spectrum of Fe 2p indicating the peaks at 711.05 eV and 724.85 eV representing Fe 2p_3/2_ and Fe 2p_1/2_, respectively, confirming the coexistence of Fe(Ⅱ) and Fe(III) in the synthesized Fe_3_O_4_@SFBC [53]. The presence of Fe(Ⅱ) and Fe(III) is attributed to the formation of Fe_2_O_3_ at 712.66 eV and FeOOH at 725.85 eV. Therefore, hydrogen bonding may have occurred during the adsorption process.

The peak of As 3d (45.25, 45.06 eV) after As(III/V) adsorption indicates that Fe_3_O_4_@SFBC has some adsorption capacity for As (Figure 12c) [54].

Figure 12d indicates the C 1s spectrum, with three main peaks, namely C-C (284 eV), C-O-C (286 eV), and O-C=O (287 eV) and C=O (288 eV) [55]. After As(III/V) adsorption, binding energies of C-C, C-O-C, and C=O reduced by 0.43/0.60/0.70 eV and 0.34/0.40/0.70 eV, respectively. The decrease in material binding energy after adsorption may be due to the formation of coordination complexes between Fe_3_O_4_@SFBC and As [56]. The results for C1s and O1s indicate the complex formation. The O atoms in C=O and C-O share an electron pair with As(III/V), thus affecting the density of the electronic cloud between the carbon and adjacent O atoms, thereby changing the binding energy [57]. Briefly, the adsorption of Fe_3_O_4_@SFBC on arsenic mainly includes electrostatic adsorption and ligand complexation.

**Figure 13:**
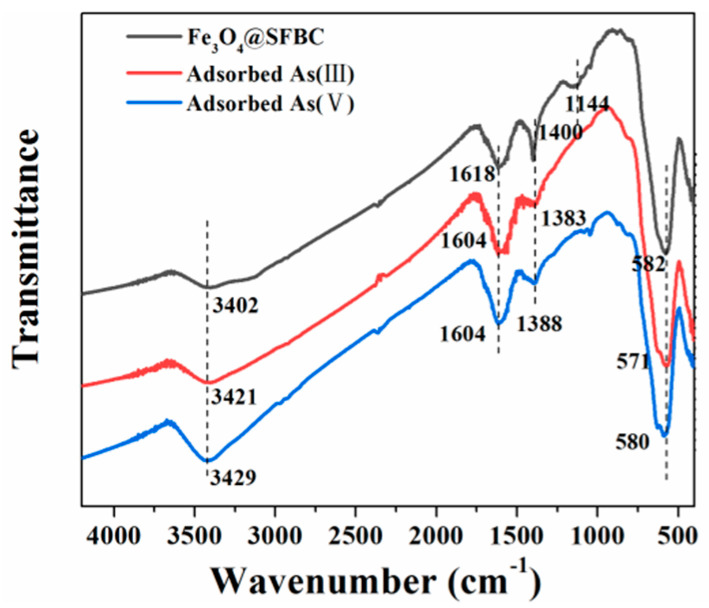
FTIR spectra of Fe_3_O_4_@SFBC before and after the adsorption of As(III/V).

To better understand the mechanism of arsenic adsorption, FTIR analysis was performed on the samples before and after the adsorption of As(III/V). Figure 13 shows that Fe_3_O_4_@SFBC has a stretching vibration peak of water at 3402 cm^−1^. However, after adsorption of As(III/V), the bend at 3421 cm^−1^ shifted to 3421 and 3429 cm^−1^, respectively, and these changes suggest that As(III/V) reacts with -OH in the protonation reaction. Moreover, the peaks in the range of 1400 cm^−1^ are significantly shifted, indicating that the vibrational correlation of C-C and -COOH is also closely related to the adsorption of As(III/V). The disappearance of the Fe-OH peak at 1144 cm^−1^ after adsorption of As(III/V) indicates that the arsenic compounds may have redox reactions with the active sites of Fe ion-related compounds during the adsorption process [58]. The main peaks after adsorption are shifted to changed degrees, representing that arsenic can be adsorbed on the surface of Fe_3_O_4_@SFBC by ion exchange, while the changes in -OH, -COOH, C-C, and Fe-OH indicate that the adsorbent experiences hydroxyl complexation on the surface [59].

#### 3.3.4. Regeneration and Reusability

**Figure 14:**
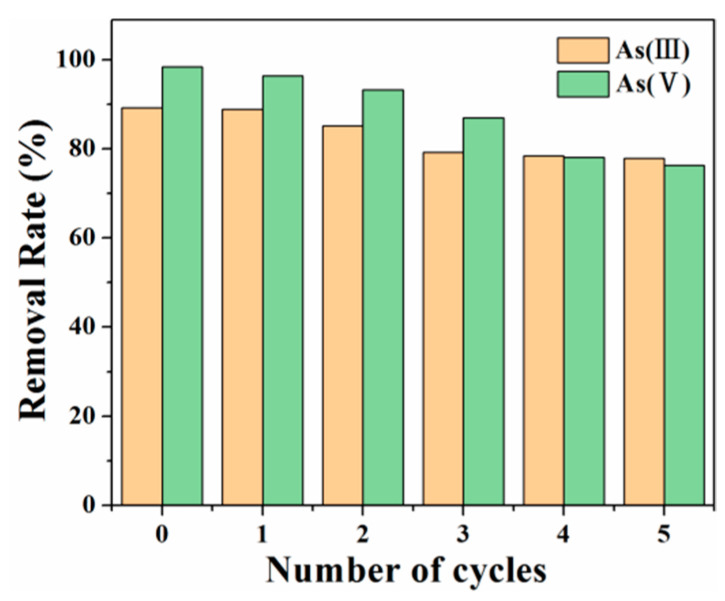
Recycling performance of different Fe_3_O_4_@SFBC materials (pH = 6 and 4, dose = 1 g/L, initial concentration = 5 mg/L, T = 25°C).

In order to ensure economic practicality, adsorption–desorption reproducibility experiments are performed on the Fe_3_O_4_@SFBC adsorbent. As(III/V) on the adsorbed Fe_3_O_4_@SFBC are desorbed with different concentrations of sodium hydroxide solution. According to the results reported by Jin et al. [60], the best desorption of As is achieved by 1 mol/L NaOH. Thus, 1 mol/L NaOH was used for desorption. The arsenic removal efficiency of the regenerated adsorbent is subjected to five consecutive cycles, as shown in Figure 14. The adsorbent exhibits good removal rates in five consecutive cycles, with As(III) and As(V) decreasing from 89.20% to 77.89% and 98.35% to 76.27%, respectively. Therefore, the adsorbent has an efficient renewal potential, and it may be effectively and continuously utilized in arsenic-contaminated wastewater treatment.

## 4. Conclusions

In this study, sunflower straw biochar was combined with Fe_3_O_4_ to prepare an efficient adsorbent for arsenic treatment. The adsorption batch experiments were carried out using Fe_3_O_4_@SFBC materials prepared calcinated at different temperatures and different SFBC/Fe mass ratios, respectively, and the results revealed that all materials conformed to the quasi-secondary kinetic equation and were consistent with the chemisorption process. As stated by the Langmuir model, the 450 °C fired SFBC showed the highest adsorption effect at pH 6 and 4 when SFBC/Fe ratio was 1:5, with maximum adsorption capacities of 121.374 and 188.753 mg/g for As(III) and As(V), respectively. Fe_3_O_4_@SFBC, compared to activated carbon materials and metal oxide composites, exhibited a stronger adsorption capacity for arsenic. The morphology and adsorption mechanism of Fe_3_O_4_@SFBC were studied, and it was found that most of the particles were 12–14 nm in size with a small size effect and good magnetization performance. Based on XPS and FTIR results, the proposed mechanism of interaction was based on chemisorption, complexation, and electrostatic interactions. Functional groups containing O, Fe, and C intrinsic to Fe_3_O_4_@SFBC played an important role in the removal of As(III/V).

Furthermore, adsorption–desorption treatment of Fe_3_O_4_@SFBC after five consecutive cycles was around 77.89% and 76.27% for As(III) and As(V) removal, respectively. Therefore, Fe_3_O_4_@SFBC can be used well as an adsorbent for arsenic removal in wastewater treatment.

## Figures and Tables

**Table 1:** Fitting parameters of adsorption kinetics.

	Object	First-Order Equation			Second-Order Equation			Conditions
		*K*_1_/min^−1^	*q_e_*/(mg/g)	*R* ^2^	*K*_2_/(g/(mg·min))	*q_e_*/(mg/g)	*R* ^2^	
Fe_3_O_4_@SFBC 350	As(III)	1.233	4.060	0.559	1.027	4.113	0.889	SFBC/Fe mass ratio 1:5
Fe_3_O_4_@SFBC 450	As(III)	1.080	4.189	0.684	0.768	4.254	0.951	
Fe_3_O_4_@SFBC 550	As(III)	1.422	4.138	0.435	1.432	4.178	0.777	
Fe_3_O_4_@SFBC 650	As(III)	1.254	4.161	0.496	1.025	4.215	0.828	
Fe_3_O_4_@SFBC 350	As(V)	1.051	4.228	0.371	0.584	4.323	0.727	
Fe_3_O_4_@SFBC 450	As(V)	1.148	4.896	0.609	0.741	4.966	0.898	
Fe_3_O_4_@SFBC 550	As(V)	1.391	4.884	0.613	1.245	4.927	0.901	
Fe_3_O_4_@SFBC 650	As(V)	1.136	4.822	0.578	0.706	4.898	0.887	
Fe_3_O_4_@SFBC 1:2	As(III)	1.851	3.772	0.280	3.427	3.791	0.603	calcination temperature of the SFBC is 450 °C
Fe_3_O_4_@SFBC 1:3	As(III)	1.323	3.763	0.447	0.979	3.821	0.808	
Fe_3_O_4_@SFBC 1:4	As(III)	1.323	3.998	0.575	1.265	4.042	0.906	
Fe_3_O_4_@SFBC 1:5	As(III)	1.080	4.189	0.684	0.768	4.254	0.951	
Fe_3_O_4_@SFBC 1:6	As(III)	1.301	3.896	0.414	1.141	3.946	0.750	
Fe_3_O_4_@SFBC 1:2	As(V)	1.319	4.627	0.450	1.006	4.684	0.819	
Fe_3_O_4_@SFBC 1:3	As(V)	1.380	4.710	0.493	1.201	4.756	0.779	
Fe_3_O_4_@SFBC 1:4	As(V)	1.670	4.879	0.447	1.986	4.909	0.820	
Fe_3_O_4_@SFBC 1:5	As(V)	1.148	4.896	0.609	0.741	4.966	0.898	
Fe_3_O_4_@SFBC 1:6	As(V)	1.644	4.824	0.386	1.844	4.857	0.746	

**Table 2:** Optimized isotherm parameters for As(III/V) adsorption by Fe_3_O_4_@SFBC magnetic nanoparticles.

	Object	Langmuir			Freundlich			Conditions
		*q_m_*/(mg/g)	*K_L_*/(L/mg)	*R* ^2^	1/*n*	*K_F_*/(m/mg)(L/mg)^1/*n*^	*R* ^2^	
Fe_3_O_4_@SFBC 350	As(III)	86.565	0.00970	0.994	0.966	0.834	0.993	SFBC/Fe mass ratio 1:5
Fe_3_O_4_@SFBC 450	As(III)	121.374	0.00759	0.999	0.960	0.944	0.999	
Fe_3_O_4_@SFBC 550	As(III)	109.061	0.00847	0.999	0.961	0.937	0.998	
Fe_3_O_4_@SFBC 650	As(III)	107.977	0.00855	0.999	0.960	0.935	0.998	
Fe_3_O_4_@SFBC 350	As(V)	138.059	0.00680	0.999	0.974	0.938	0.999	
Fe_3_O_4_@SFBC 450	As(V)	188.753	0.00542	0.999	0.971	1.041	0.999	
Fe_3_O_4_@SFBC 550	As(V)	172.638	0.00571	0.999	0.974	0.994	0.999	
Fe_3_O_4_@SFBC 650	As(V)	166.117	0.00592	0.999	0.973	0.992	0.999	
Fe_3_O_4_@SFBC 1:2	As(III)	101.761	0.00840	0.999	0.956	0.878	0.999	calcination temperature of the SFBC is 450 °C
Fe_3_O_4_@SFBC 1:3	As(III)	105.290	0.00826	0.999	0.957	0.892	0.999	
Fe_3_O_4_@SFBC 1:4	As(III)	109.382	0.00819	0.998	0.957	0.919	0.999	
Fe_3_O_4_@SFBC 1:5	As(III)	121.374	0.00759	0.999	0.960	0.944	0.999	
Fe_3_O_4_@SFBC 1:6	As(III)	116.837	0.00731	0.999	0.961	0.874	0.999	
Fe_3_O_4_@SFBC 1:2	As(V)	170.653	0.00582	0.999	0.969	1.012	0.999	
Fe_3_O_4_@SFBC 1:3	As(V)	171.149	0.00584	0.999	0.969	1.019	0.999	
Fe_3_O_4_@SFBC 1:4	As(V)	181.723	0.00561	0.999	0.970	1.039	0.999	
Fe_3_O_4_@SFBC 1:5	As(V)	188.753	0.00542	0.999	0.971	1.041	0.999	
Fe_3_O_4_@SFBC 1:6	As(V)	176.037	0.00571	0.999	0.969	1.024	0.999	

## Data Availability

The data presented in this article are available on request from the corresponding authors.
